# Factors related to curved femur in elderly Japanese women

**DOI:** 10.1080/03009734.2016.1185200

**Published:** 2016-04-23

**Authors:** Hiroyuki Tsuchie, Naohisa Miyakoshi, Yuji Kasukawa, Seietsu Senma, Yuichiro Narita, Seiya Miyamoto, Yuji Hatakeyama, Kana Sasaki, Yoichi Shimada

**Affiliations:** a Department of Orthopedic Surgery, Akita University Graduate School of Medicine, 1-1-1 Hondo, Akita 010-8543, Japan;; b Division of Orthopedic Surgery, Nakadori General Hospital, 3-15, Misono-cho, Minami-dori, Akita 010-8577, Japan

**Keywords:** 25-Hydroxyvitamin D, atypical femoral fracture, curved femur, osteomalacia, women

## Abstract

**Background:**

Multiple factors are involved in the development of atypical femoral fractures, and excessive curvature of the femur is thought to be one of them. However, the pathogenesis of femoral curvature is unknown. We evaluated the influence of factors related to bone metabolism and posture on the development of femoral curvature.

**Methods:**

A total of 139 women participated in the present study. Curvatures were measured using antero-posterior and lateral radiography of the femur. We evaluated some bone and vitamin D metabolism markers in serum, the bone mineral density (BMD), lumbar spine alignment, and pelvic tilt.

**Results:**

We divided the women into two groups, curved and non-curved groups, based on the average plus standard deviation as the cut-off between the groups. When univariate logistic regression analysis was performed to detect factors affecting femoral curvature, the following were identified as indices significantly affecting the curvature: age of the patients, serum concentrations of calcium, intact parathyroid hormone, pentosidine, homocysteine and 25-hydroxyvitamin D (25(OH)D), and BMD of the proximal femur (*P* < 0.05) both in the lateral and anterior curvatures. When we used multivariate analyses to assess these factors, only 25(OH)D and age (lateral and anterior standardized odds ratio: 0.776 and 0.385, and 2.312 and 4.472, respectively) affected the femoral curvature (*P* < 0.05).

**Conclusion:**

Femoral curvature is strongly influenced by age and serum vitamin D.

## Introduction

Although bisphosphonates (BPs) are the gold standard for osteoporosis pharmacotherapy, several adverse effects related to their long-term use have recently been reported, such as osteonecrosis of the jaw (ONJ) and atypical low-energy subtrochanteric and diaphyseal femoral fractures due to markedly suppressed bone turnover (SSBT) (1,2). These fractures are typically diagnosed as atypical femoral fractures (AFF). However, many patients have been reported with atypical femoral fractures (AFF) in the absence of BP therapy (3), although the fracture type was consistent with the criteria of AFF suggested by a task force of the American Society for Bone and Mineral Research (ASBMR) (4). The detailed cause of AFF has not been clarified, and multiple factors are thought to be involved in its development. Sasaki et al. were the first to report that excessive curvature of the femur may be one of the associated factors, and this was also stated in a review by a task force of ASBMR (4,5). However, the pathogenesis of femoral curvature in elderly women is unknown.

The aim of this study was to evaluate the influence of factors related to bone metabolism and posture on the development of femoral curvature in elderly women.

## Material and methods

### Subjects

A total of 139 women, with a mean age of 75.4 years (53 to 93), all being outpatients visiting a single institution for the treatment or examination of osteoporosis between April 2014 and March 2015, were included in this study. Of the 139 patients 64 had received osteoporosis treatment prior to evaluation: bisphosphonate, vitamin D_3_, selective estrogen receptor modulator, teriparatide, and denosumab were prescribed in 34, 19, 13, 2, and 2 patients, respectively. In addition, a history of fragility fracture was present in 29 patients: vertebral fracture, 15 patients; femoral neck or trochanteric fracture, 13 patients; and distal radius fracture, 4 patients. We excluded patients unable to walk by themselves.

### Clinical evaluations

Curvature of the right femur was measured with antero-posterior (AP) and lateral views as the angles between two linear lines drawn along the proximal and distal portions of the femoral shaft, using a method to measure femoral curvature established by Sasaki et al. (5). We performed some serum laboratory examinations of bone metabolic markers, bone quality markers, and vitamin D metabolism markers: alkaline phosphatase (ALP), calcium (Ca) adjusted for serum albumin, inorganic phosphorus (IP), intact procollagen I N-terminal propeptide (P1NP), tartrate-resistant acid phosphatase 5b (TRACP5b), pentosidine, homocysteine, intact parathyroid hormone (PTH), 25-hydroxyvitamin D (25(OH)D), and 1,25-hydroxyvitamin D_3_ (1,25(OH)_2_D). ALP, Ca, and IP were measured using standard laboratory procedures. TRACP5b was measured by an enzyme immunoassay method, P1NP and intact PTH were measured by an electrochemiluminescence immunoassay method, 25(OH)D and 1,25(OH)_2_D were measured by a radioimmunoassay method, and pentosidine and homocysteine were measured by a high-performance liquid chromatography method. We measured the bone mineral density (BMD) on AP views of the lumbar spine from L2 to L4 and the femoral neck (Discovery, Hologic Inc., MA, USA). On lateral standing X-rays of the spine including the sacrum and pelvis, we measured the lumbar lordosis angle from L1 to L5 and the pelvic inclination angle to check the lumbar spine alignment and pelvic tilt (Table 1) (6).

**Table 1.** Characteristics of study patients.

Total number	139
Age (years)	75.4 ± 10.0
AP curvature (degrees)	2.98 ± 3.32
Lateral curvature (degrees)	9.79 ± 2.48
Laboratory examinations	
ALP (IU/L)	270 ± 198
Ca (mg/dL)	9.15 ± 0.33
IP (mg/dL)	3.50 ± 0.43
P1NP (μg/L)	53.5 ± 33.5
TRACP5b (mU/dL)	280 ± 125
Pentosidine (μg/mL)	0.054 ± 0.030
Homocysteine (nmol/mL)	10.0 ± 5.9
Intact PTH (pg/mL)	44.0 ± 20.2
25(OH)D (ng/mL)	22.6 ± 8.3
1,25(OH)_2_D (pg/mL)	51.1 ± 18.6
BMD (g/cm^2^)	
Lumbar spine	0.722 ± 0.146
Proximal femur	0.486 ± 0.114
Lumbar lordosis angle (degrees)	23.1 ± 18.6
Lumbo-sacral angle (degrees)	25.6 ± 10.0
Steroid usage: number	5/139
Average	4.4 ± 3.4
Vitamin D usage: number	19/139

Values are expressed as number of patients, or mean ± SD with ranges.

1,25(OH)_2_D: 1,25-hydroxyvitamin D_3_; 25(OH)D: 25-hydroxyvitamin D; ALP: alkaline phosphatase; AP: antero-posterior; BMD: bone mineral density; Ca: calcium; IP: inorganic phosphorus; P1NP: intact procollagen I N-terminal propeptide; PTH: parathyroid hormone; TRACP5b: tartrate-resistant acid phosphatase 5b.

### Statistical analysis

All values are expressed as the mean ± standard deviation (SD). We divided the women into two groups, curved and non-curved groups, based on the average plus standard deviation as the cut-off between the groups for lateral and anterior curvature respectively: the lateral curvature was 6.3 degrees and anterior curvature was 12.3 degrees. Because there are many evaluation items, we extracted some factors likely to have associations with femoral curvature by univariate logistic regression. Multivariate logistic regression analysis was used to examine the factors of femoral curvature. Probability (*P*) values less than 0.05 were considered statistically significant.

## Results

For lateral curvature, the curved group included 25 patients with a mean age of 82.7 years, and the non-curved group included 114 patients with a mean age of 73.8 years. For anterior curvature, the curved group included 19 patients with a mean age of 84.9 years, and the non-curved included 120 patients with a mean age of 73.9 years. The number of patients who were non-curved based on both definitions was 107, the number of patients who were curved based on the lateral but not anterior curvature definition was 13, the number of patients who were curved based on the anterior but not lateral curvature definition was 7, and the number of patients who were curved based on both lateral and anterior curvature definitions was 12.

When univariate logistic regression analysis was performed to identify factors affecting femoral curvature we found that the age of the patients, serum concentrations of Ca, intact PTH, pentosidine, and 25(OH)D, and BMD of the proximal femur, both with regard to lateral (Table 2) and anterior curvatures (Table 3), differed between women belonging to the non-curved and curved groups. Homocysteine, however, only did so with regard to the lateral curvature (Table 2). We used multivariate logistic regression analysis to exclude a mutual influence of different factors, and then only 25(OH)D and age affected the femoral curvature (Tables 4 and 5).

**Table 2.** Univariate logistic regression analysis of lateral curvature.

Variables	Non-curved	Curved	Standardized OR	95% CI	*P*
Number	114	25			
Age (years)	73.8 ± 9.8	82.7 ± 6.9	3.263	1.767–6.024	<0.001
Laboratory examinations					
ALP (IU/L)	268 ± 212	279 ± 117	1.053	0.768–1.567	0.797
Ca (mg/dL)	9.18 ± 0.31	9 ± 0.39	0.563	0.352–0.9	0.017
IP (mg/dL)	3.52 ± 0.43	3.45 ± 0.44	0.848	0.542–1.325	0.468
P1NP (μg/L)	53.3 ± 33.5	54 ± 34.2	1.019	0.662–1.57	0.93
TRACP5b (mU/dL)	280 ± 123	280 ± 141	1	0.648–1.541	0.999
Pentosidine (μg/mL)	0.049 ± 0.02	0.077 ± 0.053	2.158	1.363–3.416	0.001
Homocysteine (nmol/mL)	9.0 ± 3.4	14.3 ± 11	2.606	1.479–4.592	<0.001
Intact PTH (pg/mL)	41.6 ± 18	55.0 ± 25.9	1.792	1.197–2.682	0.005
25(OH)D (ng/mL)	24 ± 8	16.4 ± 6.5	0.263	0.135–0.512	<0.001
1,25(OH)_2_D (pg/mL)	51.9 ± 19.1	47.4 ± 16.1	0.767	0.475–1.239	0.279
BMD (g/cm^2^)					
Lumbar spine	0.73 ± 0.144	0.683 ± 0.155	0.715	0.452–1.13	0.151
Proximal femur	0.503 ± 0.109	0.411 ± 0.106	0.433	0.269–0.697	<0.001
Lumbar lordosis angle (degrees)	23.4 ± 19.1	21.4 ± 16.3	0.891	0.582–1.364	0.62
Lumbo-sacral angle (degrees)	25.8 ± 10.5	25.0 ± 7.5	0.93	0.604–1.43	0.74
Steroid usage (mg)	0.04 ± 0.26	0.72 ± 2.25	11.53	0.918–144.8	0.058
Vitamin D usage (number)	16	3	0.835	0.224–3.117	0.958

Values are expressed as number of patients, or mean ± SD with ranges.

1,25(OH)_2_D: 1,25-hydroxyvitamin D_3_; 25(OH)D: 25-hydroxyvitamin D; 95% CI: 95% confidence interval; ALP: alkaline phosphatase; BMD: bone mineral density; Ca: calcium; IP: inorganic phosphorus; OR: odds ratio; P1NP: intact procollagen I N-terminal propeptide; PTH: intact parathyroid hormone; TRACP5b: tartrate-resistant acid phosphatase 5b.

**Table 3.** Univariate logistic regression analysis of anterior curvature.

Variables	Non-curved	Curved	Standardized OR	95% CI	*P*
Number	120	19			
Age (years)	73.9 ± 9.7	84.9 ± 5.4	5.413	2.391–12.26	<0.001
Laboratory examinations					
ALP (IU/L)	252 ± 135	381 ± 404	1.546	0.990–2.415	0.055
Ca (mg/dL)	9.18 ± 0.31	9 ± 0.43	0.573	0.342–0.962	0.036
IP (mg/dL)	3.52 ± 0.43	3.41 ± 0.44	0.754	0.451–1.258	0.279
P1NP (μg/L)	53.3 ± 33.1	54.6 ± 37.2	1.04	0.644–1.68	0.872
TRACP5b (mU/dL)	279 ± 120	285 ± 159	1.045	0.648–1.686	0.856
Pentosidine (μg/mL)	0.05 ± 0.02	0.082 ± 0.058	2.168	1.361–3.452	0.001
Homocysteine (nmol/mL)	9.6 ± 5.8	11.9 ± 6.4	1.317	0.895–1.938	0.163
Intact PTH (pg/mL)	42.2 ± 19.4	54.1 ± 23	1.633	1.064–2.506	0.025
25(OH)D (ng/mL)	23.6 ± 8.1	16.8 ± 6.6	0.328	0.166–0.649	0.001
1,25(OH)_2_D (pg/mL)	51.3 ± 19.1	49.3 ± 15.2	0.893	0.537–1.486	0.189
BMD (g/cm^2^)					
Lumbar spine	0.728 ± 0.149	0.679 ± 0.121	0.698	0.418–1.167	0.171
Proximal femur	0.497 ± 0.113	0.422 ± 0.097	0.517	0.313–0.851	0.01
Lumbar lordosis angle (degrees)	23.0 ± 18.2	23.2 ± 21.3	0.998	0.617–1.613	0.979
Lumbo-sacral angle (degrees)	25.7 ± 10.1	24.9 ± 9.6	0.921	0.57–1.488	0.737
Steroid usage (mg)	0.16 ± 1	0	–	–	−
Vitamin D usage (number)	17	2	0.713	0.151–3.367	0.943

Values are expressed as number of patients, or mean ± SD with ranges.

1,25(OH)_2_D: 1,25-hydroxyvitamin D_3_; 25(OH)D: 25-hydroxyvitamin D; 95% CI: 95% confidence interval; ALP: alkaline phosphatase; BMD: bone mineral density; Ca: calcium; IP: inorganic phosphorus; OR: odds ratio; P1NP: intact procollagen I N-terminal propeptide; PTH: intact parathyroid hormone; TRACP5b: tartrate-resistant acid phosphatase 5b.

**Table 4.** Multivariate logistic regression analysis of lateral curvature.

Variables	Standardized OR	95% CI	*P*
Age (years)	2.312	1.142–4.681	0.02
Laboratory examinations			
Ca (mg/dL)	0.649	0.344–1.223	0.187
Pentosidine (μg/mL)	1.445	0.833–2.509	0.193
Homocysteine (nmol/mL)	1.202	0.749–1.928	0.444
Intact PTH (pg/mL)	1.209	0.713–2.050	0.479
25(OH)D (ng/mL)	0.776	0.426–1.414	0.004
BMD (g/cm^2^)			
Proximal femur	0.776	0.426–1.414	0.404

Values are expressed as number of patients, or mean ± SD with ranges.

25(OH)D: 25-hydroxyvitamin D; 95% CI: 95% confidence interval; BMD: bone mineral density; Ca: calcium; OR: odds ratio; PTH: parathyroid hormone.

**Table 5.** Multivariate logistic regression analysis of anterior curvature.

Variables	Standardized OR	95% CI	*P*
Age (years)	4.472	1.770–11.3	0.002
Laboratory examinations			
Ca (mg/dL)	0.65	0.331–1.278	0.187
Pentosidine (μg/mL)	1.551	0.926–2.596	0.096
Intact PTH (pg/mL)	1.145	0.658–1.994	0.632
25(OH)D (ng/mL)	0.385	0.165–0.898	0.027
BMD (g/cm^2^)			
Proximal femur	1.111	0.564–2.19	0.763

Values are expressed as number of patients, or mean ± SD with ranges.

25(OH)D: 25-hydroxyvitamin D; 95% CI: 95% confidence interval; BMD: bone mineral density; Ca: calcium; OR: odds ratio; PTH: parathyroid hormone.

## Discussion

We evaluated some factors that may affect the development of femoral curvature, such as the bone turnover, bone quality, vitamin D metabolism, BMD, and posture, and only 25(OH)D and the age were selected as indices significantly affecting the curvature. Osteomalacia is a condition causing the deposition failure of calcium and phosphorus in osteoids after epiphyseal line closure. The causes are broadly divided into failure of vitamin D action and hypophosphatemia; failure of vitamin D action includes the deficiency of vitamin D, abnormal vitamin D metabolism, and abnormalities of the vitamin D receptor (7). Published data indicate that the presence of vitamin D deficiency should be suspected when serum 25(OH)D is lower than 20 ng/mL (8,9). In this study, curved group patients with both lateral and anterior curvature showed low serum 25(OH)D, at about 16 ng/mL. Although there is a report that vitamin D becomes lower as age increases (10), the serum 25(OH)D concentrations showed significant correlations with femoral curvature both anteriorly and laterally in the present study, even when we eliminated the effect of age. It might be anticipated that vitamin D deficiency, namely osteomalacia, strongly affects the curvature of the femur.

The ASBMR Task Force 2013 Revised Case Definition of AFF is as follows: 1) the fracture is associated with minimal or no trauma, as in a fall from a standing height or lower; 2) the fracture line originates at the lateral cortex and is markedly transverse in its orientation, although it may become oblique as it progresses medially across the femur; 3) complete fracture extends through both cortices and may be associated with a medial spike, whereas incomplete fracture only involves the lateral cortex; 4) the fracture is not comminuted or is minimally comminuted; and 5) localized periosteal or endosteal thickening of the lateral cortex is present at the fracture site (‘beaking’ or ‘flaring’) (4). We previously reported a case of osteomalacia with marked femoral curvature being consistent with the AFF definition (11). Although plain radiographs showed areas of endosteal thickening and horizontal lines resembling fractures over the outer cortical bones of the femoral diaphysis in this case, these constituted a Looser zone, a conventional X-ray sign of osteomalacia. Osteomalacia not only affects the femoral curvature, considered as one of the causes of AFF, but it may also have been present in previously reported AFF cases with femoral curvature.

Saita et al. reported that the fracture sites of AFF are associated with weight-bearing lower limb alignment (12), and we assumed that posture also affected it. Spino-pelvic alignment of lumbar kyphosis with posterior pelvic tilt on standing requires hip joint extension, knee joint flexion, and ankle joint dorsiflexion to maintain postural balance. Thus, the femur is positioned obliquely to the ground, not vertically, and excessive muscular force on the thigh may be required. Although we considered that an increased load on the thigh might cause a curved femur, we could not show any relation between the curved femur and posture change, such as lumbar kyphosis and pelvic tilt.

Bone quality markers, such as pentosidine and homocysteine, can be used to evaluate the deterioration of bone collagen indirectly (13). Deterioration of bone collagen causes a deterioration of bone quality, and the bone strength also declines. We suspected that bone quality affects femoral curvature, but we were unable to demonstrate such a relation. Saita et al. indicated that steroid use may be related to AFF (14), and we could also consider the influence of bone quality. Although there was no relation between the presence of curved femur and steroid usage in this study (Tables 2 and 3), we could not sufficiently evaluate this because steroid users only numbered 5 people. We have to perform further detailed studies with a larger number of patients treated with steroids.

In conclusion, to the best of our knowledge, the present study is the first to examine the influence of factors related to bone metabolism and posture on the development of femoral curvature in elderly women. Such curvatures were strongly influenced by low serum vitamin D concentrations. Further extended and detailed studies have to be performed in order to clarify the relation between AFF and femoral curvature.
